# Thymoquinone Protects Against Cardiac Hypertrophy via PPAR‐γ/PI3K/Akt Pathway

**DOI:** 10.1111/jcmm.70911

**Published:** 2026-01-30

**Authors:** Rong‐bin Qiu, Zi‐ming Wu, Zhi‐qiang Xu, Li‐juan Hu, Shi‐tao Zhao, Rui‐yuan Zeng, Zhi‐cong Qiu, Lian‐fen Zhou, Song‐qing Lai, Wen‐jun Wang, Li Wan

**Affiliations:** ^1^ Department of Cardiac Surgery The First Affiliated Hospital of Nanchang University Nanchang China; ^2^ Institute of Cardiovascular Surgical Diseases, Jiangxi Academy of Clinical Medical Sciences The First Affiliated Hospital of Nanchang University Nanchang China; ^3^ Gannan Health Vocational College Ganzhou China

**Keywords:** apoptosis, cardiac hypertrophy, ferroptosis, PI3K/Akt, PPAR‐γ, thymoquinone

## Abstract

Thymoquinone (TQ), the principal active constituent of Nigella stativa, has demonstrated numerous biological properties and therapeutic effects on various diseases. However, its therapeutic potential against cardiac hypertrophy remains uncertain. This study aims to investigate the protective effects of TQ on stress‐induced cardiac hypertrophy and elucidate the underlying mechanisms. Our findings reveal that TQ mitigates stress‐induced cardiac hypertrophy in mice and AngII‐induced hypertrophy in H9c2 cells. Moreover, TQ inhibits cardiomyocyte ferroptosis and apoptosis by downregulating PTGS2, Bax, and upregulating GPX4, Bcl‐2, thereby alleviating cardiac hypertrophy and dysfunction. Mechanistically, the protective effects of TQ against ferroptosis and apoptosis in cardiac hypertrophy were reversed by the PPAR‐γ inhibitor (GW9662). In addition, TQ treatment led to increased protein expression levels of P‐PI3K and P‐AKt. Taken together, our findings suggest that TQ could attenuate cardiac hypertrophy through activation of the PPAR‐γ/PI3K/Akt signalling pathway.

## 1 Introduction

The primary function of the heart is to ensure adequate perfusion of surrounding organs. Under conditions of prolonged pressure overload, the myocardium increases in mass, contractile force is enhanced, and cardiomyocytes enlarge, a condition known as myocardial hypertrophy [1]. Cardiac hypertrophy is generally categorised into physiological and pathological hypertrophy. Physiological hypertrophy is characterised by the absence of significant fibrosis and cell death, is reversible, and does not progress to heart failure. In contrast, pathological myocardial hypertrophy poses several risks, including ventricular arrhythmia and sudden cardiac death [2]. Persistent cardiac hypertrophy can result in myocardial fibrosis, ventricular remodelling, heart failure, and ultimately, death [3, 4]. The underlying mechanisms of pathological cardiac hypertrophy include mitochondrial dysfunction, cardiomyocyte death, and increased reactive oxygen species (ROS) production [1]. Excessive ROS production in cardiomyocytes leads to mitochondrial dysfunction. Thus, regulating ROS levels and maintaining their dynamic balance can effectively prevent cardiac hypertrophy [5]. Persistent cardiac hypertrophy has severe societal implications, including significant economic burdens [6]. Therefore, actively seeking prevention and treatment strategies for cardiac hypertrophy is imperative.

Increasing evidence indicates that cardiomyocyte ferroptosis plays a role in the development of cardiac hypertrophy [1, 7]. Ferroptosis is a distinct form of cell death, differing morphologically, biochemically, and genetically from apoptosis and other types of necrosis [8]. Ferroptosis is primarily characterised by its iron dependency and lipid peroxidation, distinguishing it from apoptosis, necrosis, and autophagy [9]. Studies have shown that ROS play a crucial role in cardiovascular diseases [10]. Excessive ROS production in cardiomyocytes leads to lipid and protein oxidation, resulting in single‐stranded DNA breaks and inducing cardiac hypertrophy [11]. During ferroptosis, cardiomyocyte mitochondria typically exhibit structural changes, such as outer mitochondrial membrane rupture, increased membrane density, and loss of cristae [12]. Furthermore, regulatory proteins like GPX4 and FSP1 are involved in ferroptosis regulation by affecting iron metabolism or clearing lipid peroxidation products. Studies have confirmed that GPX4 is a key protein in ferroptosis [13]. Tomonori Tadokoro found that upregulating GPX4 protein expression can inhibit ferroptosis and alleviate cardiac dysfunction [14]. Additionally, ferroptosis in cardiomyocytes activates fibroblasts, inducing an immune response that increases collagen secretion, leading to myocardial fibrosis, hypertrophy, and heart failure [15]. Clearly, ferroptosis is a crucial mechanism in the occurrence and progression of cardiovascular diseases.

*Nigella sativa* is a ranunculaceae herb, predominantly found in regions like Yunnan and Xinjiang in China. Its seeds are frequently used in traditional medicine to treat various ailments, including gastrointestinal diseases, headaches, infections, hypertension, bronchial asthma, and eczema [16, 17]. TQ is a natural bioactive compound extracted and purified from the seeds of *Nigella sativa* , with the molecular formula C10H12O2 [18]. TQ exhibits antioxidant, anti‐inflammatory, immunomodulatory, antibacterial, and anti‐tumour properties, providing protective effects against damage to the heart, brain, liver, and kidneys [19]. Numerous studies have confirmed that TQ regulates the biological behaviour of various tumour cells, including those in colon cancer [20], breast cancer [21], lung cancer [22] and prostate cancer [23]. However, evidence of TQ's therapeutic effects on cardiovascular diseases remains limited.

Accumulating evidence indicates that the PI3K/Akt signalling pathway plays a pi votal role in the pathogenesis of cardiovascular diseases [24, 25]. However, the precise role of the PPAR‐γ/PI3K/Akt pathway in mediating the protective effects of TQ remains elusive. The present study was designed to investigate the impact of TQ on cardiac hypertrophy and to elucidate the underlying mechanism of the PPAR‐γ/PI3K/Akt pathway in the cardioprotective effects of TQ. Consequently, this study has the potential to identify a novel therapeutic target and treatment strategy for the clinical management of cardiac hypertrophy.

## 2 Materials and Methods

### 2.1 Reagents and Chemicals

TQ (purity ≥ 99.59%), Ferrostatin‐1 (Fer‐1, ferroptosis inhibitor, purity ≥ 99.96%), and GW9662 (purity ≥ 99.87%) were purchased from MedChemExpress (MCE). Angiotensin II was purchased from GLPBIO. Phalloidin was obtained from UElandy (UE). Primary antibodies against P‐PI3K (cat no. 310164), PI3K (cat no. 251221), P‐Akt (cat no. 310021), Akt (cat no. R23412), and secondary antibodies goat anti‐mouse (cat no. 511103) and goat anti‐rabbit (cat no. 511203) were obtained from Chengdu Zhengneng Biotechnology Co. Ltd. Collagen I (cat no. 14695‐1‐AP), GPX4 (cat no. 30388‐1‐AP), PTGS2 (cat no. 27308‐1‐AP), Bcl2 (cat. no. 26593–1‐AP), BAX (cat. no. 50599‐2‐Ig), ANP (cat no. 27426–1‐AP), and β‐actin (cat. no. 66009‐1‐Ig) were sourced from Proteintech Group Inc. PPAR‐γ (cat. no. YT3836) was acquired from ImmunoWay Biotechnology Company. BNP (cat. no. A2179) was sourced from ABclonal Biotechnology Co. Ltd.

### 2.2 Cell Model of Cardiac Hypertrophy and Treatment

The rat H9c2 cell line was purchased from the Cell Bank of the Chinese Academy of Sciences. Cells were cultured as a monolayer in high‐glucose Dulbecco's modified Eagle's medium (H‐DMEM; HyClone, Cytiva) supplemented with 10% fetal bovine serum (FBS; Gibco, Thermo Fisher Scientific Inc.) and 1% penicillin–streptomycin (Gibco‐BRL) at 37°C under standard conditions (95% humidity, 21% O_2_, and 5% CO_2_) [26]. The H9c2 cells were then divided into six different treatment groups as follows:

1. Control group: H9c2 cells were cultured in H‐DMEM for 48 h under standard conditions
2. AngII group: H9c2 cells were exposed to 1 μM AngII for 24 h after being cultured for 24 h under standard conditions [25].
3. AngII + TQ group: H9c2 cells were treated with 10 μM TQ for 24 h, then cultured with 1 μM AngII for another 24 h
4. AngII + Fer‐1 group: H9c2 cells were incubated with 10 μM Fer‐1 for 2 h, then treated with 1 μM AngII for another 24 h.
5. AngII + TQ + PPAR‐γ antagonist (GW9662) group: H9c2 cells were incubated with 10 μM TQ and 10 μM PPAR‐γ antagonist (GW9662) for 24 h, then treated with 1 μM AngII for another 24 h

### 2.3 Cell Viability Assay

Cell viability was assessed using the Cell Counting Kit‐8 (CCK‐8; GlpBio Technology). H9c2 cells were seeded in 96‐well plates at a density of 2 × 10^4^ cells/well and treated with TQ at concentrations of 1, 5, 10, and 20 μM for 24 h. Then, 10 μL of CCK‐8 reagent was added to each well. Finally, the absorbance was measured at 450 nm using a microplate reader (Thermo Fisher Scientific Inc.).

### 2.4 Observation of Cell Size via Phalloidin Staining

Dissolve the dye in sterile water and dilute it to the appropriate concentration according to the instructions in the kit. After processing the cells, wash them three times with PBS. Then, fix the cells in the paraformaldehyde fixative at room temperature for 20 min. Subsequently, permeabilize the cells with 0.4% Triton X—100 for 10 min. Next, add the diluted Rhodamine‐Phalloidin working solution for staining, and observe the samples under a fluorescence microscope.

### 2.5 Determination Levels of ROS

ROS production levels were detected using dichlorofluorescein diacetate (DCFH‐DA, Beyotime Institute of Biotechnology) staining. H9c2 cells were incubated with 10 μM DCFH‐DA for 20 min at 37°C in the dark, then washed three times with H‐DMEM without 10% FBS. Lastly, intracellular ROS levels were analyzed using a fluorescence microscope (magnification, ×200).

### 2.6 Measurement of Malondialdehyde (MDA), Glutathione (GSH) and Glutathione Disulfide (GSSG)

The Malondialdehyde (MDA) Assay Kit, Glutathione (GSH) Assay Kit, and Glutathione Disulfide (GSSG) Assay Kit (all from Beyotime Institute of Biotechnology) were used to measure MDA, GSH, and GSSG levels in H9c2 cells, following the manufacturer's instructions. The ratio of reduced glutathione (GSH) to oxidised glutathione (GSSG) was also determined.

### 2.7 FerroOrange Staining

Intracellular Fe^2+^ levels were assessed using FerroOrange (Dojindo Laboratories Inc.) according to the manufacturer's instructions. Briefly, H9c2 cardiomyocytes treated with 1 μM FerroOrange were incubated at 37°C for 30 min in the dark. Excess FerroOrange was removed by washing the cells twice with PBS. Finally, fluorescence microscopy (200× magnification) was used to assess ferrous iron levels.

### 2.8 Western Blot Analysis

Total proteins were extracted from H9c2 cells and mouse myocardial tissue using RIPA lysis buffer (Beijing Solarbio Science & Technology Co. Ltd.). Protein concentration was quantified using the BCA Assay Kit (Good Laboratory Practice Bioscience). Equal amounts of total protein (30–40 μg) were separated by 10%–12.5% gradient SDS‐PAGE and transferred to polyvinylidene fluoride (PVDF) membranes (PALL Corporation). The PVDF membranes were blocked with 5% skimmed milk for 2 h at room temperature and then incubated overnight at 4°C with specific primary antibodies against GPX4 (1:1000), PTGS2 (1:1000), Bax (1:1000), Bcl‐2 (1:1000), collagen I (1:1000), PPAR‐γ (1:500), P‐PI3K (1:500), P‐Akt (1:500), PI3K (1:500), Akt (1:500), ANP (1:800), BNP (1:800), and β‐actin (1:1000) on a shaker. The membranes were then washed three times and incubated with HRP‐conjugated secondary antibodies for 2 h at room temperature. β‐actin was used as an internal control. Finally, the optical density of the protein bands was quantified using ImageJ software (National Institutes of Health).

### 2.9 RNA Isolation and Quantitative Real‐Time PCR

qRT‐PCR was used to evaluate the mRNA expression levels of ANP, BNP, and collagen I. Total RNA was extracted using TRIzol reagent (Beijing Solarbio Science & Technology Co. Ltd.). Total RNA was reverse transcribed using the PrimeScript RT reagent kit (Monad Biotech Co. Ltd.). The primers used are described in Table 1.

**TABLE 1:** Primers used for of real‐time RT‐PCR.

Gene	Species	Forward primer (5′ → 3′)	Reverse primer (5′ → 3′)
ANP	Rat	ATCTGATGGATTTCAAGAACC	CTCTGAGACGGGTTGACTTC
BNP	Rat	ACAATCCACGATGCAGAAGCT	GGGCCTTGGTCCTTTGAGA
ANP	Mouse	CTCCGATAGATCTGCCCTCTTGAA	GGTACCGGAAGCTGTTGCAGCCTA
BNP	Mouse	GCTCTTGAAGGACCAAGGCCTCAC	GATCCGATCCGGTCTATCTTGTGC
Collagen I	Rat	GAGAGAGCATGACCGATGGATT	TGGACATTAGGCGCAGGAA
Collagen I	Mouse	AGGCTTCAGTGGTTTGGATG	CACCAACAGCACCATCGTTA
ACTB	Mouse	TGACAGGATGCAGAAGGAGA	GTACTTGCGCTCAGGAGGAG
ACTB	Rat	AGATGACCCAGATCATGTTTGAGA	CGCTCGGTCAGGATCTTCAT

### 2.10 Animal of Cardiac Hypertrophy and Treatment

The experimental procedures adhered strictly to the guidelines provided by the National Institutes of Health (NIH) and the ARRIVE (Animals in Research: Reporting In Vivo Experiments) guidelines and received approval from the Animal Experimentation Ethics Committee of First hospital of Nanchang University (CDYFY‐IACUC‐202407QR115). C57BL/6J mice aged 8–10 weeks and weighing 20–24 g, sourced from Changzhou Cavens Model Animal Co. Ltd., were randomly divided into four groups, consisting of six mice per group: a sham operation group, a TAC operation group, a TAC + TQ treatment group, and a TAC + TQ + GW9662 (PPAR‐γ antagonist) group. Cardiac hypertrophy was induced in mice via transverse aortic constriction (TAC) surgery, as previously described in a study [27]. Mice in the TQ treatment group received TQ (50 mg/kg, dissolved in corn oil) via gavage for 6 weeks post‐TAC surgery [28]. Mice in the PPAR‐γ antagonist group received intraperitoneal injections of GW9662 (1 mg/kg) three times a week for 6 weeks post‐operation. Echocardiography was performed prior to euthanasia to assess myocardial hypertrophy and evaluate the efficacy of TQ treatment. Euthanasia was achieved by administering an overdose of sodium pentobarbital (200 mg/kg, i.p).

### 2.11 Transthoracic Echocardiography

Mice were initially anaesthetised with an intraperitoneal injection of 2% pentobarbital (45 mg/Kg). The Vevo2100 imaging system (VisualSonics Inc.) was used to assess left ventricular ejection fraction (EF), left ventricular fractional shortening (FS), left ventricular internal dimension in diastole (LVIDd), and left ventricular posterior wall dimension (LVPWd) using 2‐dimensional transthoracic echocardiography.

### 2.12 Histological Analysis

Mouse heart weight (HW) and body weight (BW) were measured. Heart specimens were fixed in formalin, paraffin‐embedded according to previous studies [29], and the wax blocks were cut into 5 μm sections and stained with haematoxylin and eosin and Masson's trichrome staining according to standard methods [30, 31]. The size of myocardial cells was determined by analysing ventricular slices stained with fluorescein isothiocyanate (FITC)‐labelled wheat germ agglutinin (WGA).

### 2.13 TUNEL Assay

Terminal deoxynucleotidyl transferase dUTP nick‐end labeling (TUNEL) staining was conducted to quantify apoptotic cells in vivo using a commercial kit (Roche). Cardiac tissue sections were prepared and stained according to the manufacturer's protocol. Apoptotic cells, characterised by apoptotic nuclei centrally located, were visualised using a Nikon fluorescent microscope (Nikon Eclipse Ti‐SR, Tokyo, Japan).

### 2.14 Statistical Analysis

Statistical analysis was performed using GraphPad Prism 9.0 software. One‐way ANOVA with Tukey post hoc test was performed to compare data between multiple groups. A value of *p* < 0.05 was considered statistically significant.

## 3 Results

### 3.1 Effects of TQ on the Survival of H9c2 Cell

**FIGURE 1:**
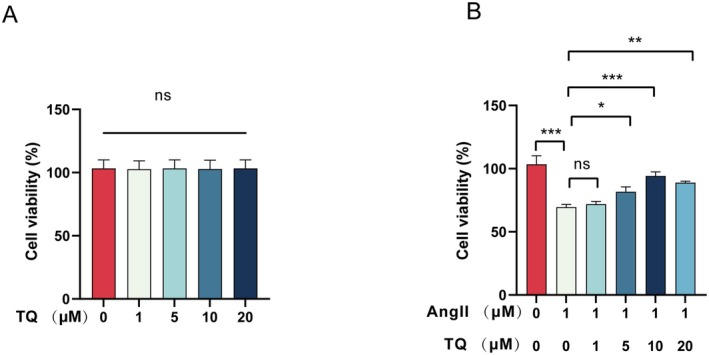
Effects of TQ on the survival of H9C2 cells. (A) The viability of H9C2 cells was assessed following treatment with varying concentrations of TQ (0, 1, 5, 10, 20 μM). (B) The viability of H9C2 cells was assessed following treatment with varying concentrations of TQ and 1 μM AngII. Data are presented as mean ± SD. *p < 0.05, **p < 0.01, ***p < 0.001, ns, not significant.

The viability of H9c2 cells treated with various concentrations of TQ (0, 1, 5, 10, 20 μM) and angiotensin II (AngII) (1 μM) was evaluated using the Cell Counting Kit‐8 (CCK‐8) assay. No significant differences in cell viability were observed after treating the cells with different TQ concentrations compared to the control group (Figure 1A). However, H9c2 cells stimulated with AngII (1 μM) exhibited a significant reduction in viability, which was subsequently restored by pretreatment with TQ (Figure 1B). Given that the 10 μM concentration demonstrated the most substantial protective effect on cell viability, it was chosen for further experiments.

### 3.2 TQ Attenuated Cardiomyocyte Hypertrophy In Vivo and In Vitro

**FIGURE 2:**
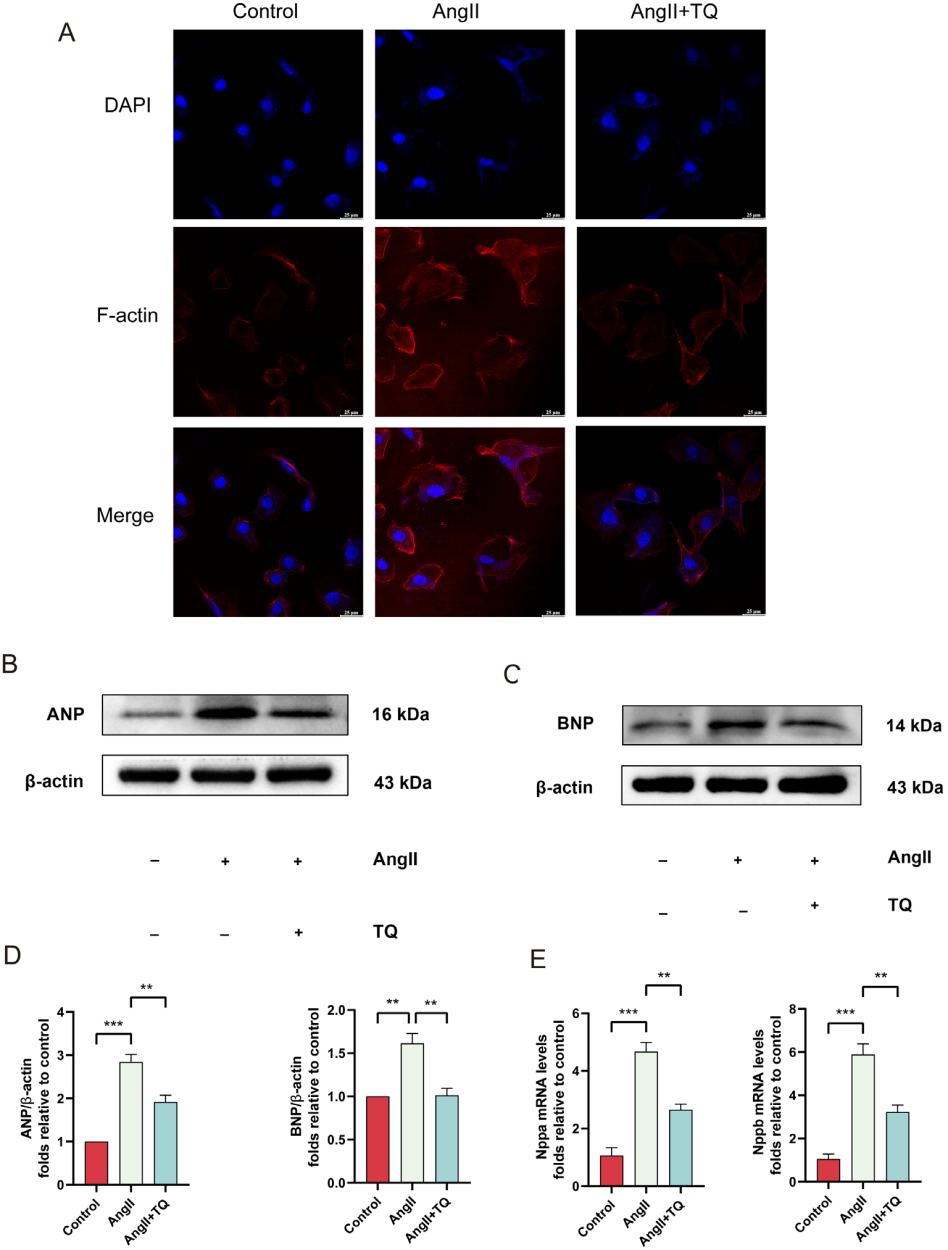
TQ attenuated cardiomyocyte hypertrophy in vitro. (A) Images of H9C2 cell stained for Phalloidin Staining (red) (scale bar, 25 μm). (B–D) Immunoblotting analysis of ANP and BNP protein expression levels in vivo, along with quantification of the immunoblotting results. β‐actin was used as an internal control. (E) The mRNA expression levels of the myocardial hypertrophy genes ANP and BNP were detected by real‐time quantitative PCR (qPCR). Data are presented as mean ± SD. **p < 0.01, ***p < 0.001.

To investigate the protective effect of TQ against AngII‐induced hypertrophy in H9C2 cells, we pretreated H9C2 cells with AngII (1 μM) for 24 h to induce hypertrophy and assessed cell surface area using Phalloidin Staining. Our findings indicated that pretreatment with TQ significantly reduced cell surface area (Figure 2A). Additionally, we measured protein and mRNA expression levels of key cardiac hypertrophy markers, including ANP and BNP (Figure 2B–E). TQ pretreatment significantly attenuated the expression of ANP and BNP. Collectively, these results suggest that TQ effectively mitigates AngII‐induced hypertrophy in H9C2 cells.

**FIGURE 3:**
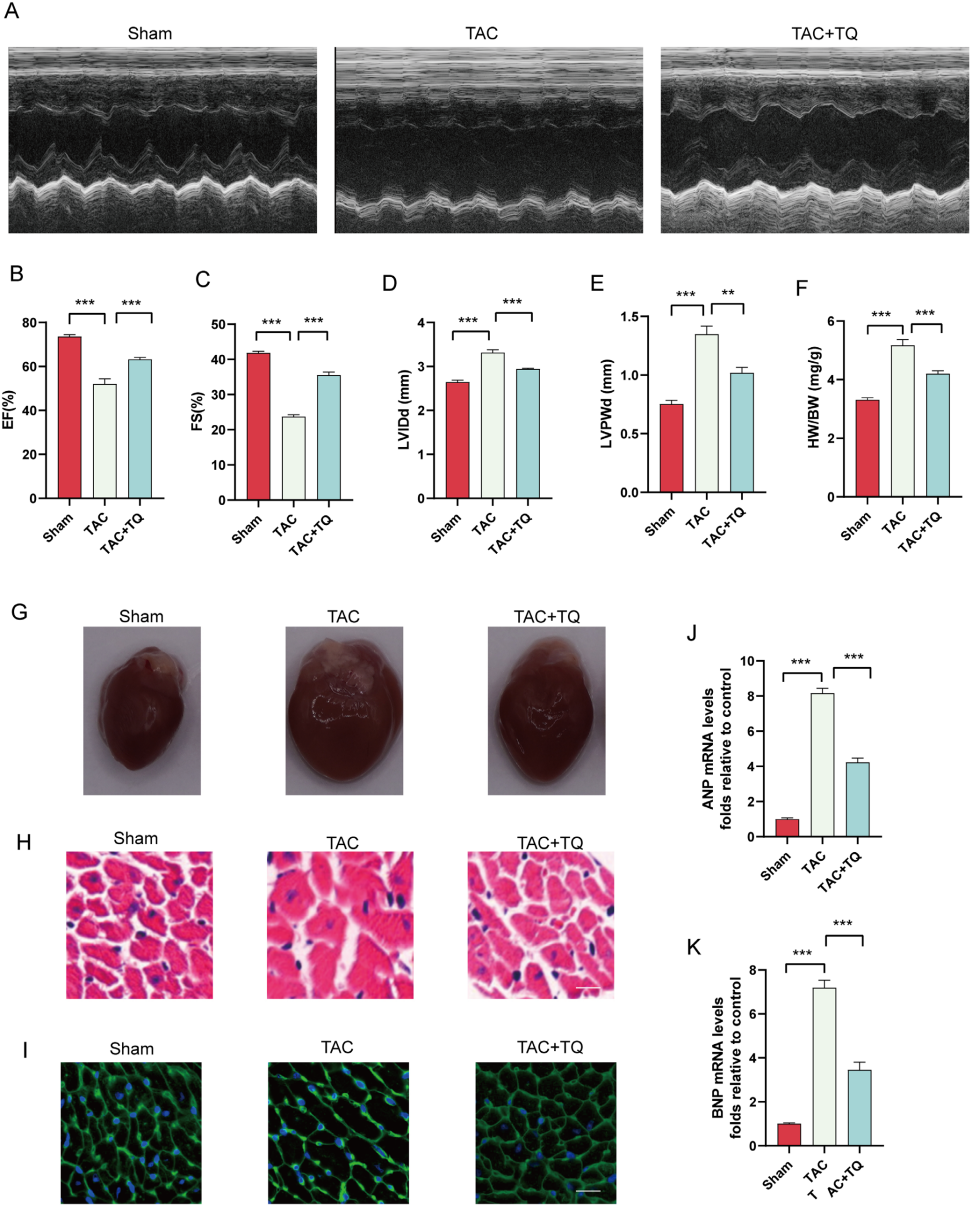
TQ attenuated cardiomyocyte hypertrophy in vivo. (A) Echocardiography was conducted to assess heart function. (B–E) EF (ejection fraction), FS (fractional shortening of the left ventricular diameter), LVIDd (diastolic left ventricular internal diameter), LVPWd (diastolic left ventricular posterior wall thickness). (F) Ratios of heart to body weight. (G) The gross morphology of the cardiac tissue. (H) Haematoxylin and eosin‐stained cardiac cross‐sections (magnification, ×200; scale bar, 100 μm). (I) Histological assessment of cardiac hypertrophy utilising FITC‐wheat germ agglutinin (WGA) staining (magnification, ×200; scale bar, 50 μm). (J, K) The effects of TQ on the mRNA expression of hypertrophic markers ANP, BNP. Data are presented as mean ± SD, **p < 0.01, ***p < 0.001.

To further demonstrate the cardioprotective effects of TQ on cardiac hypertrophy, we established a pressure overload hypertrophy model via transverse aortic constriction (TAC) surgery. Cardiac function was evaluated using echocardiography (Figure 3A). Mice in the TAC group exhibited reduced left ventricular ejection fraction (EF), decreased left ventricular fractional shortening (FS), increased left ventricular internal dimension diastole (LVIDd), and left ventricular posterior wall dimension (LVPWd) compared to the sham group (Figure 3B–E). However, post‐TAC TQ treatment improved cardiac function and mitigated cardiac hypertrophy (Figure 3A–E). Additionally, the heart weight/body weight (HW/BW) ratio was lower in the TQ group compared to the TAC group (Figure 3F). Pretreatment with TQ reduced TAC‐induced cardiac hypertrophy, as evidenced by decreased cardiac size (Figure 3G). Haematoxylin and eosin staining revealed significant heart size increase and myofibril degeneration in TAC group mice, whereas TQ‐treated mice showed reduced heart size and improved myofibril integrity (Figure 3H). Subsequently, we quantified the average cross‐sectional area of cardiomyocytes utilising wheat germ agglutinin (WGA) staining. Notably, 6 weeks post‐transverse aortic constriction (TAC) surgery, the cross‐sectional area of myocytes exhibited a significant increase when compared to the sham group. However, the administration of TQ reversed the change (Figure 3I). Moreover, ANP and BNP expression levels were significantly downregulated in the TQ group compared to the TAC group (Figure 3J,K). In conclusion, TQ can attenuate pressure overload‐induced cardiac hypertrophy.

### 3.3 TQ Inhibited Cardiac Fibrosis In Vivo and In Vitro

**FIGURE 4:**
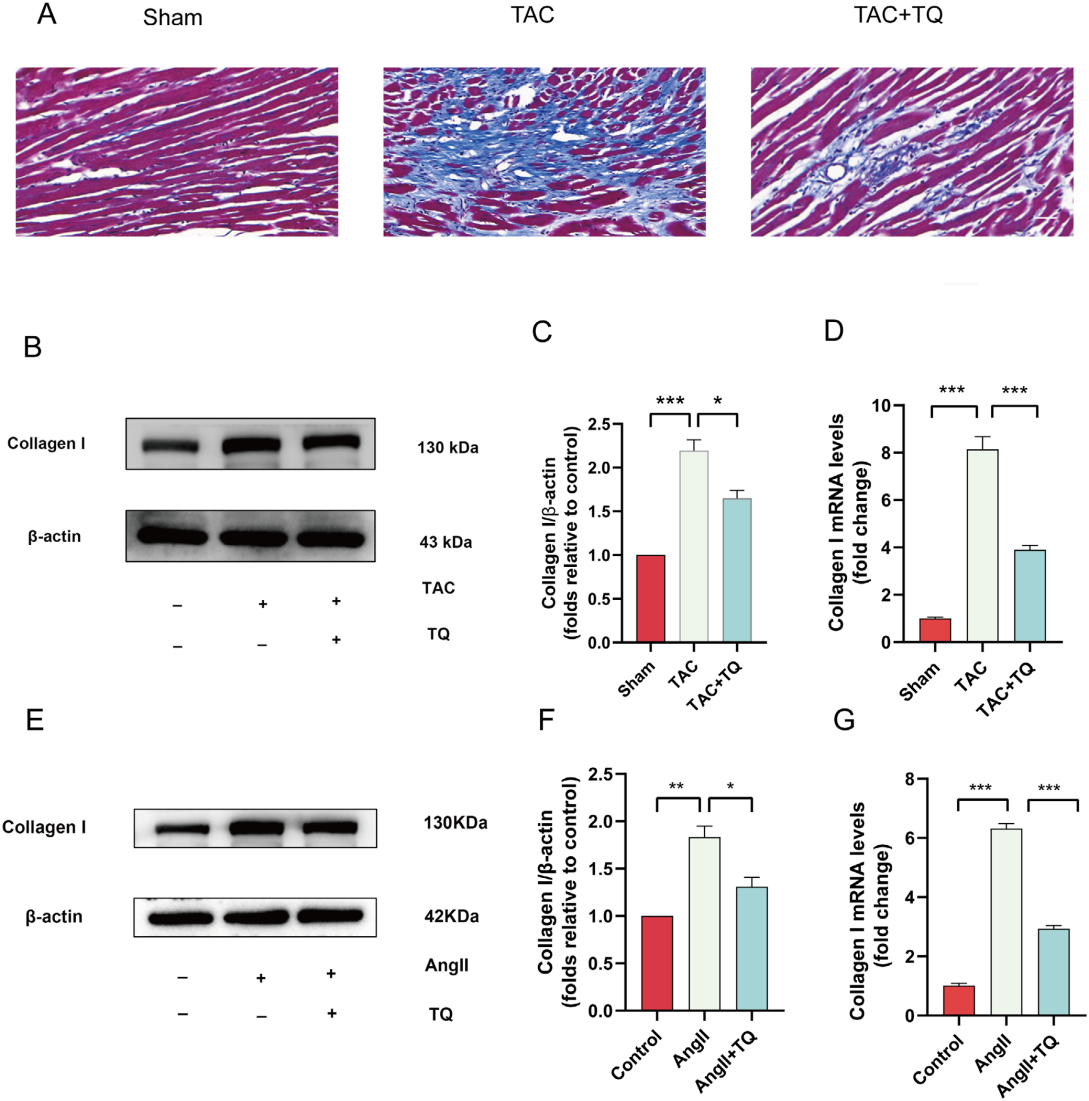
TQ inhibited cardiac fibrosis in vivo and in vitro. (A) Masson's trichrome staining for cardiac fibrosis (magnification, ×100; scale bar, 200 μm). (B, C) Immunoblotting analysis of collagen I protein expression levels in vivo, along with quantification of the immunoblotting results. β‐actin was used as an internal control. (D) The effects of TQ on collagen I mRNA expression in vivo were assessed using qRT‐PCR. (E, F) Immunoblotting analysis of collagen I protein expression levels in vitro, along with quantification of the immunoblotting results. β‐actin was used as an internal control. (G) The impact of TQ on the expression of collagen I mRNA in vitro was evaluated through the utilisation of qRT‐PCR. Data are presented as mean ± SD. *p < 0.05, **p < 0.01, ***p < 0.001.

Studies have shown that cardiac fibrosis and cardiac hypertrophy coexist and mutually promote each other. As fibrosis progresses, it impairs the heart's contractile function, leading to heart failure and potentially fatal outcomes [32, 33]. We used Masson staining, immunoblotting, and RT‐qPCR to evaluate the effects of TQ on cardiac fibrosis. Masson staining revealed a significant increase in fibrosis in mice subjected to TAC surgery (Figure 4A). Compared to the control group, the TAC group showed significantly elevated protein and mRNA expression levels of type I collagen, which were reduced in the TQ group (Figure 4B–D). Our in vitro findings mirrored the in vivo results, with AngII treatment increasing protein and mRNA expression of type I collagen, while TQ treatment decreased these levels (Figure 4E–G).

### 3.4 TQ Alleviates AngII‐Induced Hypertrophy in H9C2 Cells Through Inhibiting Ferroptosis

**FIGURE 5:**
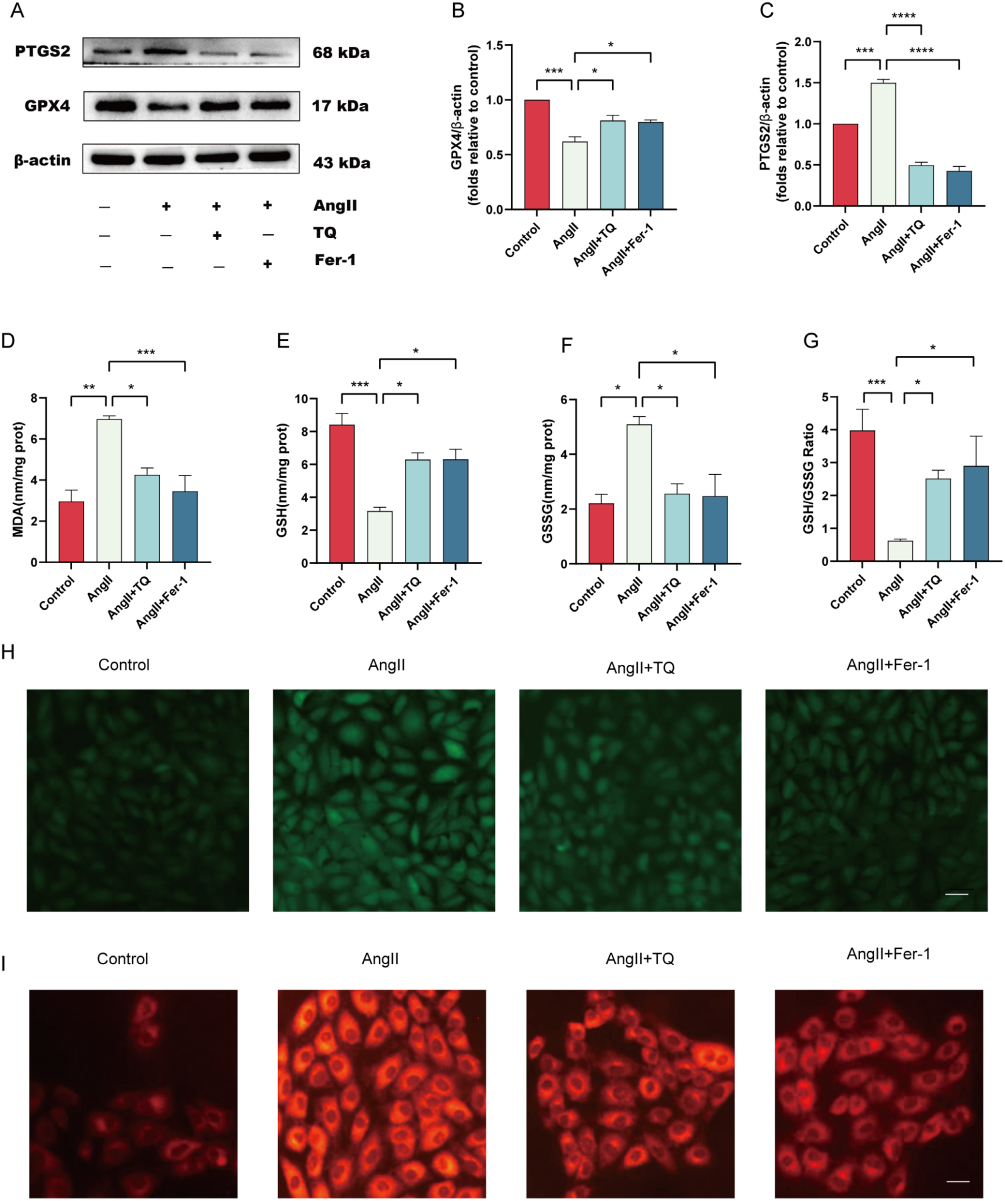
TQ alleviates AngII‐induced hypertrophy in H9C2 cells through inhibiting ferroptosis. (A–C) Immunoblotting analysis of PTGS2 and GPX4 protein expression levels in vitro, along with quantification of the immunoblotting results. β‐actin was used as an internal control. The levels of (D) MDA, (E) GSH, (F) GSSG, and (G) GSH/GSSG ratios were quantified using appropriate kits in AngII‐induced H9c2 cells following treatment with TQ and Fer‐1. (H) Images stained with DCFH‐DA for ROS detection in H9C2 cells (magnification: ×200; Scale bar: 100 μm). (I) Intracellular Fe2+ levels were assessed using FerroOrange in AngII‐induced cells following treatment with TQ and Fer‐1 (magnification, ×200; scale bar, 100 μm). Data are presented as mean ± SD. *p < 0.05, **p < 0.01, ***p < 0.001, ****p < 0.0001.

Various studies have underscored the critical role of ferroptosis in the pathogenesis of cardiac hypertrophy, showing that inhibition of ferroptosis effectively mitigates the progression from cardiac hypertrophy to heart failure. To validate the inhibitory effects of TQ on ferroptosis and its potential in attenuating cardiac hypertrophy, we assessed the expression levels of key ferroptosis marker proteins GPX4 and PTGS2 [34]. Our findings demonstrated that pretreatment with TQ and ferrostatin‐1 (Fer‐1) significantly upregulated GPX4 protein expression while downregulating PTGS2 protein expression compared to the AngII group (Figure 5A–C). Accumulation of intracellular iron ions and lipid peroxidation are recognised as crucial mechanisms underlying ferroptosis [35, 36]. Levels of malondialdehyde (MDA), a lipid peroxidation product, were significantly elevated in the AngII group compared to controls. However, pretreatment with TQ and Fer‐1 resulted in decreased levels of these markers (Figure 5D). Additionally, we quantified levels of glutathione (GSH), oxidised glutathione (GSSG), and the GSH/GSSG ratio in H9c2 cells. Our results showed that AngII‐induced H9c2 cells exhibited elevated GSSG levels, accompanied by reduced GSH levels and GSH/GSSG ratio. Importantly, these alterations were effectively reversed by pretreatment with TQ and Fer‐1 (Figure 5E–G). The production of large amounts of reactive oxygen species (ROS) can cause abnormal lipid metabolism and lead to lipid peroxidation, which is a hallmark of ferroptosis [37]. Our results indicated that ROS levels were significantly increased in the AngII group; however, pretreatment with TQ and Fer‐1 reduced ROS levels (Figure 5H). When cells undergo ferroptosis, the excessive accumulation of divalent iron ions in cells triggers the Fenton reaction and the generation of abundant ROS, which leads to an increase in the level of intracellular oxidative stress. The results in Figure 5 I show that the fluorescence intensity was significantly increased in the AngII‐induced H9C2 cell model group. Interestingly, pretreatment with TQ and Fer‐1 significantly reversed this increase in fluorescence intensity. In conclusion, our study has demonstrated that ferroptosis plays a role in angiotensin II (AngII)‐induced hypertrophy in H9C2 cells, and the protective effect exerted by TQ is mediated through the modulation of ferroptosis.

### 3.5 TQ Suppresses Ferroptosis in Cardiac Hypertrophy Through Upregulating PPAR‐γ

**FIGURE 6:**
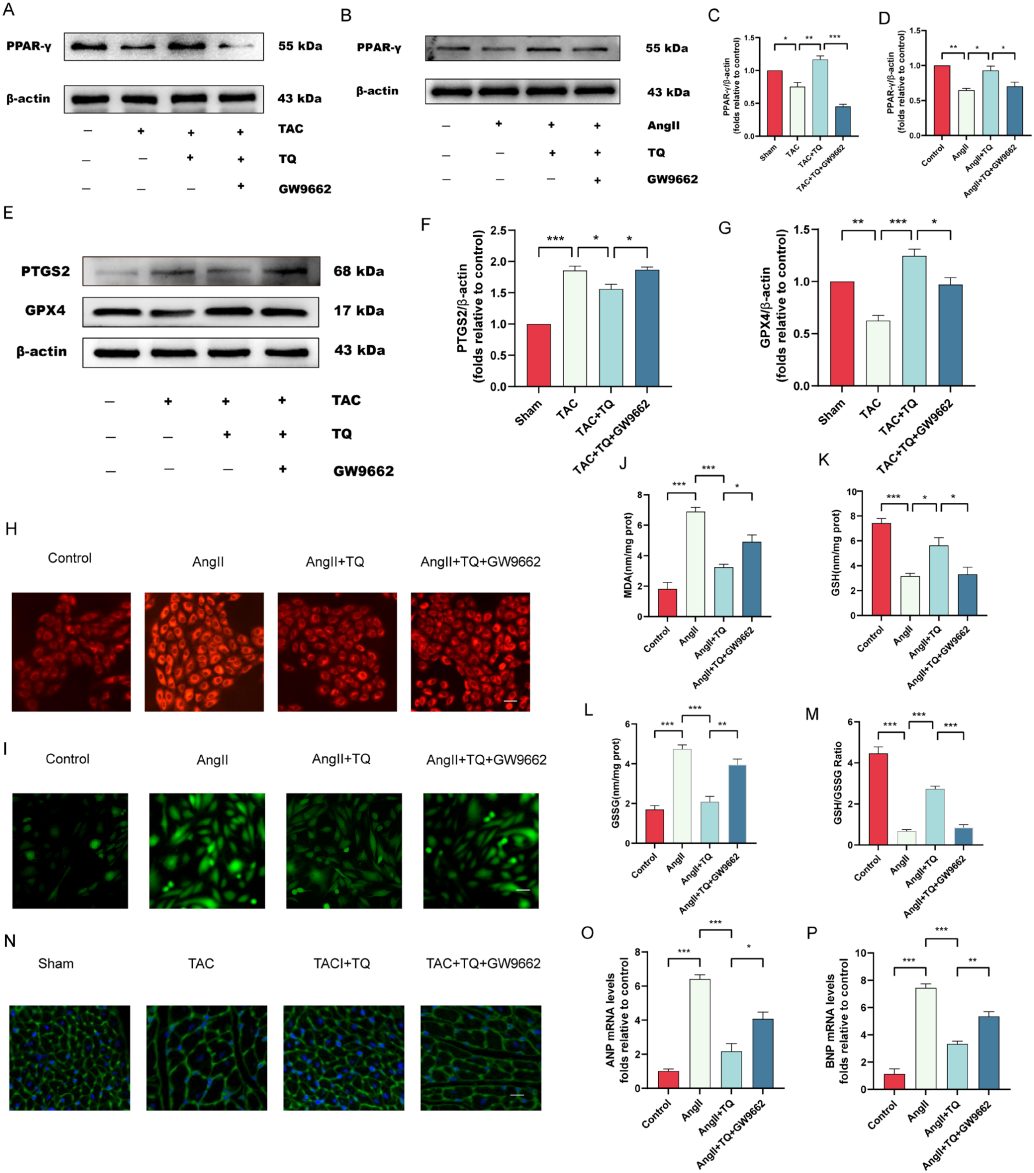
TQ suppresses ferroptosis in cardiac hypertrophy through upregulating PPAR‐γ. (A–D) Immunoblotting analysis of PPAR‐γ protein expression levels in vivo and in vitro, along with quantification of the immunoblotting results. β‐actin was used as an internal control. (E–G) Immunoblotting analysis of PTGS2 and GPX4 protein expression levels in vivo and in vitro following treatment with TQ, GW9662, along with quantification of the immunoblotting results. β‐actin was used as an internal control. (H) Intracellular Fe2+ levels were assessed using FerroOrange in AngII‐induced cells following treatment with TQ, GW9662 (magnification, ×200; scale bar, 100 μm). (I) Images stained with DCFH‐DA for ROS detection in H9C2 cells (magnification: ×200; scale bar: 100 μm). The levels of (J) MDA, (K) GSH, (L) GSSG, and (M) GSH/GSSG ratios were quantified using appropriate kits in AngII‐induced H9c2 cells following treatment with TQ, GW9662. (N) Histological assessment of cardiac hypertrophy utilising FITC‐wheat germ agglutinin (WGA) staining (magnification, ×200; scale bar, 50 μm). (O, P) The effects of TQ on the mRNA expression of hypertrophic markers ANP and BNP. Data are presented as mean ± SD. *p < 0.05, **p < 0.01, ***p < 0.001.

Peroxisome proliferator‐activated receptor γ (PPAR‐γ) is a protective regulator essential for maintaining cardiac homeostasis and preventing heart failure [38, 39, 40]. To validate the inhibitory effect of TQ on ferroptosis through PPAR‐γ regulation in cardiac hypertrophy, we employed a PPAR‐γ inhibitor (GW9662) to inhibit PPAR‐γ activity. To confirm whether PPAR‐γ is involved in the cardioprotective effects of TQ pretreatment, we evaluated its protein expression levels. Western blot analysis revealed that TQ upregulated PPAR‐γ expression in both in vivo and in vitro experiments. However, when GW9662 was used to inhibit the activity of PPAR‐γ, the aforementioned changes were significantly reversed (Figure 6A–D). Additionally, we measured the protein expression levels of PTGS2 and GPX4 in vivo, finding that PTGS2 expression was significantly increased and GPX4 expression was decreased in the TAC and AngII groups. TQ pretreatment reversed these changes, but the protective effect of TQ was significantly negated by GW9662 pretreatment (Figure 6E–G). To further verify that TQ inhibits ferroptosis by upregulating PPAR‐γ, we assessed MDA, ferrous iron content, ROS levels, GSH, GSSG, and the GSH/GSSG ratio. Our results showed that TQ pretreatment decreased MDA, ferrous iron content, ROS levels, and GSSG, while increasing GSH and the GSH/GSSG ratio. When the activity of PPAR‐γ was inhibited, the aforementioned trend was reversed (Figure 6H–M). To further investigate the impact of TQ on myocardial hypertrophy through the upregulation of PPAR‐γ and the inhibition of ferroptosis, we employed GW9662. WGA staining revealed that the reduction in cardiomyocyte area induced by TQ was reversed by GW9662 (Figure 6N). Furthermore, GW9662 restored the downregulated mRNA levels of hypertrophy markers, ANP and BNP, in the TQ‐treated group (Figure 6O,P).

### 3.6 TQ Inhibits Apoptosis in Cardiac Hypertrophy Through Upregulating PPAR‐γ

**FIGURE 7:**
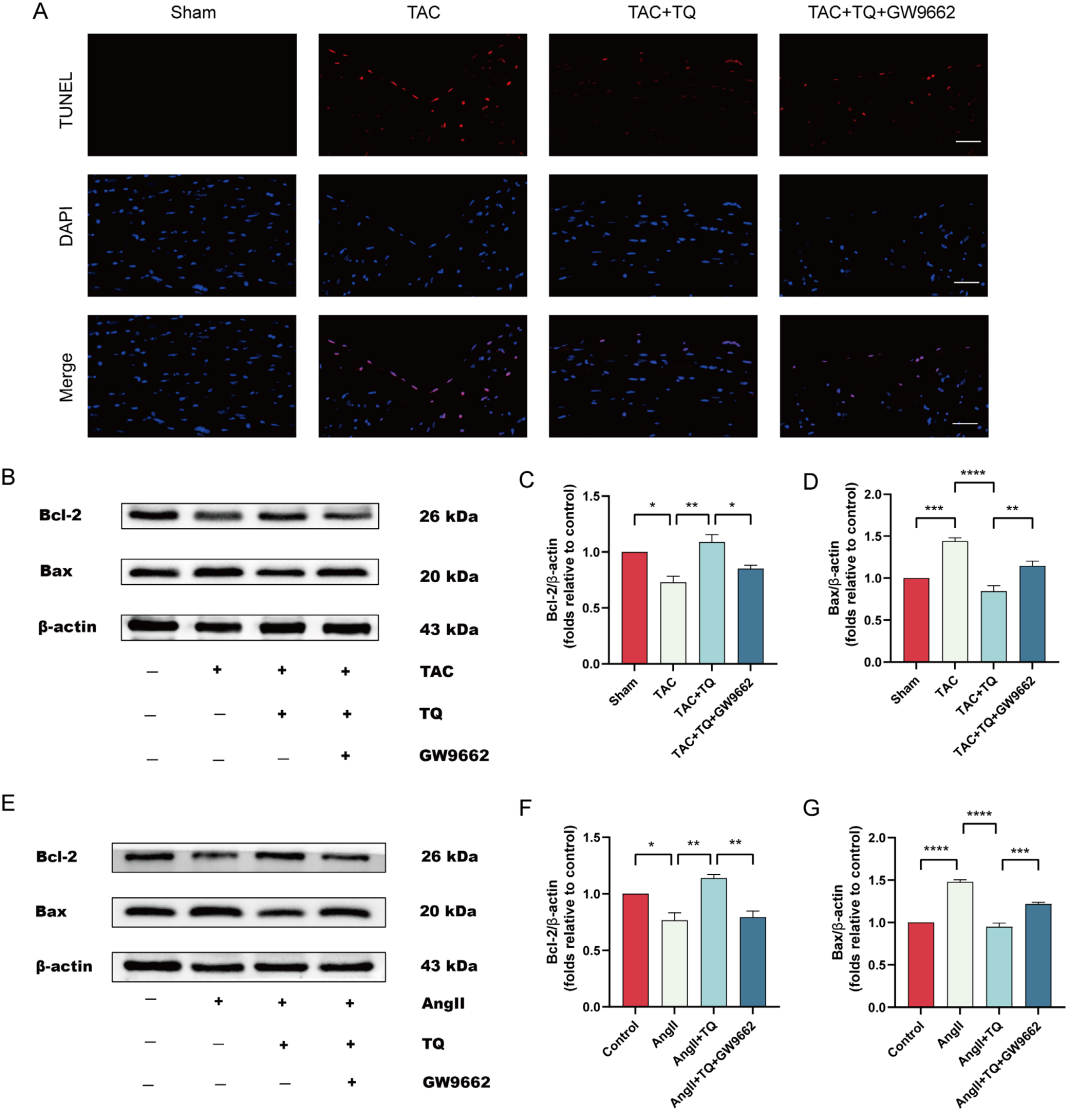
TQ inhibits apoptosis in cardiac hypertrophy through upregulating PPAR‐γ (A) TUNEL staining was performed in cardiac tissues (magnification, x200; scale bar, 50 μm). (B–D) Immunoblotting analysis of Bcl‐2 and Bax protein expression levels in vivo following treatment with TQ, GW9662, along with quantification of the immunoblotting results. β‐actin was used as an internal control. (E–G) Immunoblotting analysis of Bcl‐2 and Bax protein expression levels in vitro following treatment with TQ, GW9662, along with quantification of the immunoblotting results. β‐actin was used as an internal control. Data are presented as mean ± SD. *p < 0.05, **p < 0.01, ***p < 0.001, ****p < 0.0001.

Apoptosis plays a crucial role in the pathogenesis of cardiac hypertrophy and heart failure [41]. Under long‐term stress conditions, apoptosis can lead to myocardial cell damage and energy deficiency, eventually resulting in heart failure [42]. The proportion of apoptotic cells increased in TAC‐operated mice, as depicted in the figure, and decreased following TQ treatment. Notably, inhibition of PPAR‐γ activity reversed the protective effect of TQ (Figure 7A). Western blot analysis further validated TQ's inhibitory impact on apoptosis, showing that TQ upregulated Bcl‐2 protein levels while downregulating Bax protein levels. Conversely, inhibition of PPAR‐γ activity resulted in downregulated Bcl‐2 protein levels and upregulated Bax protein levels (Figure 7B–D). Similar results were obtained in AngII‐treated H9c2 cells in vitro (Figure 7E–G). Collectively, these findings suggest that TQ exhibits a protective effect by suppressing apoptosis through the regulation of the PPAR‐γ signalling pathway.

### 3.7 Protective Effects of TQ Through the PPAR‐γ/P I3K/AKT Signalling Pathway

**FIGURE 8:**
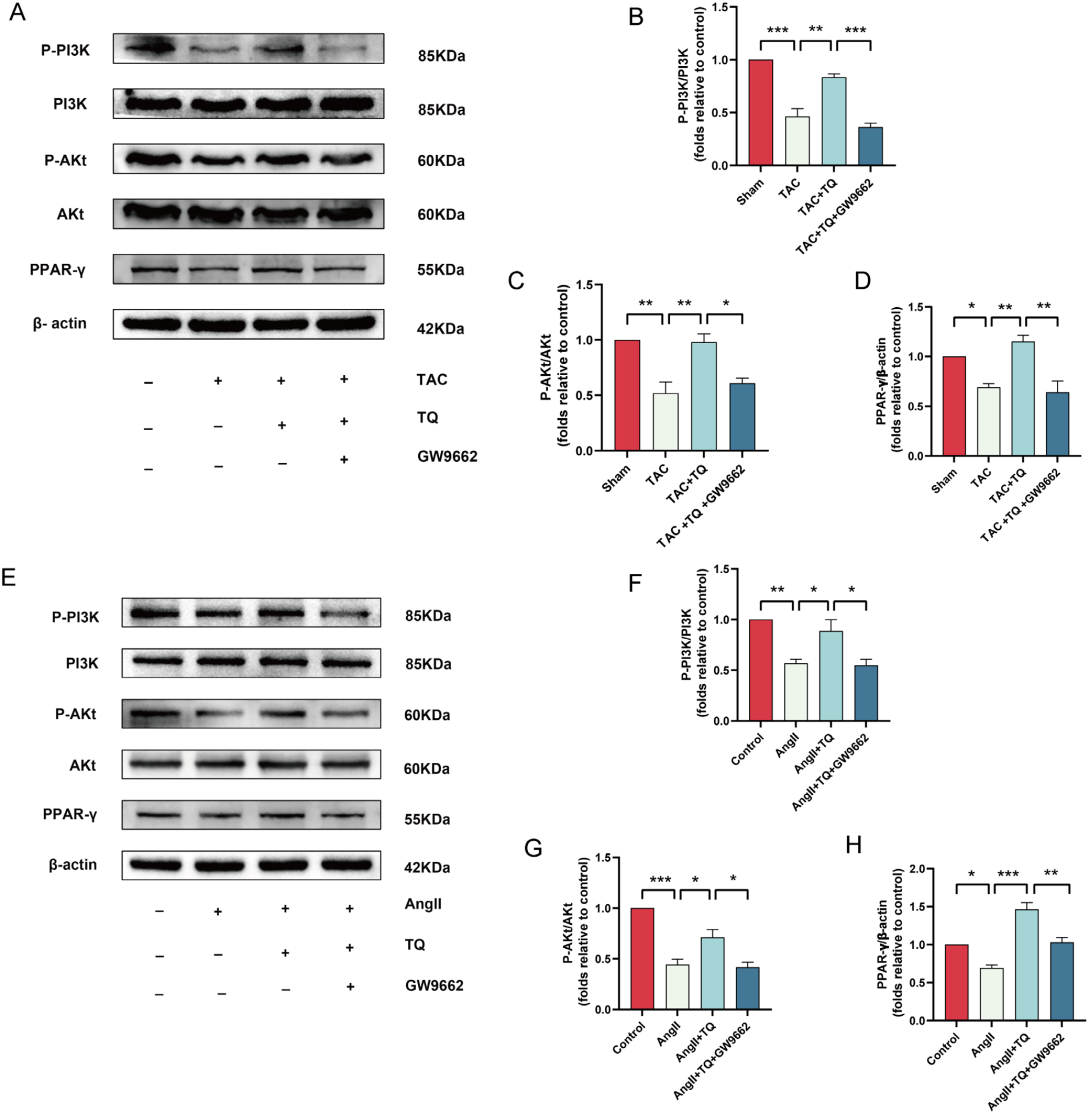
Protective effects of TQ through the PPAR‐γ/PI3K/AKT signalling pathway (A–D) Immunoblotting analysis of P‐PI3K, PI3K, P‐AKT, AKT, PPAR‐γ protein expression levels in vivo, along with quantification of the immunoblotting results. β‐actin was used as an internal control. (E–H) Immunoblotting analysis of P‐PI3K, PI3K, P‐AKT, AKT, PPAR‐γ protein expression levels in vitro, along with quantification of the immunoblotting results. β‐actin was used as an internal control. Data are presented as mean ± SD. *p < 0.05, **p < 0.01, ***p < 0.001.

The aforementioned studies indicate that TQ has the potential to mitigate cardiac hypertrophy by inhibiting ferroptosis and apoptosis, achieved through the up‐regulation of PPAR‐γ expression. However, the precise role of the PPAR‐γ/PI3K/Akt pathway in the cardioprotective effects of TQ remains unclear. To clarify the involvement of the PPAR‐γ/PI3K/Akt pathway in the protective effects of TQ, we conducted Western blot analysis to detect the phosphorylation levels of PI3K and Akt. Our results indicated that PI3K and Akt phosphorylation were decreased following transverse aortic constriction (TAC) treatment but increased after TQ pretreatment; this effect was reversed by GW9662 (Figure 8A–D). Furthermore, TQ pretreatment enhanced PI3K and Akt phosphorylation in angiotensin II (AngII)‐treated H9C2 cells, and this enhancement was also reversed by GW9662 (Figure 8E–H). Based on these findings, we propose that the PPAR‐γ/PI3K/Akt pathway is involed in the cardioprotective effects of TQ.

## 4 Discussion

Myocardial hypertrophy is an adaptive response to physiological and pathological overload, characterised by an increase in the surface area of cardiomyocytes [43]. It is a significant factor in the occurrence and progression of many heart diseases. Persistent cardiac hypertrophy can lead to structural cardiac disorders, resulting in systolic dysfunction, heart failure, arrhythmia, and sudden death [44]. Since no effective treatment exists to reduce cardiac hypertrophy, a deep understanding of the underlying molecular mechanisms is essential for developing new drugs for the prevention and treatment of heart failure. In this study, we reveal that TQ attenuates TAC‐induced cardiac hypertrophy in mice in vivo and AngII‐induced hypertrophy in H9c2 cells in vitro. We found that TQ inhibited myocardial fibrosis, reduced ROS production, and inhibited ferroptosis and apoptosis by upregulating PPAR‐γ. In addition, the present study mainly revealed that TQ could protect against cardiac hypertrophy through activation of the PPAR‐γ/PI3K/Akt pathway. This finding offers new insights into the therapeutic potential of TQ in treating pathological cardiac hypertrophy.

In recent years, the clinical application prospects of seeds from Melanaceae species have garnered increasing attention. TQ, a natural active monomer extracted and purified from the seeds of black cumin ( *Nigella sativa* ), exhibits antioxidant, anti‐inflammatory, and anti‐tumour properties [17]. Numerous studies have confirmed that TQ regulates the proliferation and apoptosis of tumour cells and exerts inhibitory effects on various tumours, including colorectal cancer [45], pancreatic cancer [46], breast cancer [47], and prostate cancer [23]. However, the role of TQ in alleviating myocardial hypertrophy has been seldom studied. In this study, we found that TQ has a protective effect on cardiac hypertrophy. Our results show that TQ has the potential to alleviate cardiac hypertrophy, as evidenced by a reduction in the surface area of cardiomyocytes observed through WGA staining, as well as a decrease in the mRNA expression levels of hypertrophic genes ANP and BNP, detected via qPCR. Thus, we have identified a new therapeutic direction for TQ.

Increasing evidence has shown that ferroptosis is involved in the regulation of myocardial hypertrophy, which is one of the pathological mechanisms of heart failure. Inhibiting ferroptosis to reduce myocardial hypertrophy plays a crucial role in the prevention and treatment of heart failure [15]. Wang et al. [48] found that depletion of Mixed Lineage Kinase 3 (MLK3), a member of the MAP3K family, could inhibit ferroptosis and attenuate TAC‐induced cardiac hypertrophy. The primary mechanism by which cold stress leads to cardiac hypertrophy, cardiac remodelling, and myocardial contractility impairment is ferroptosis in cardiomyocytes. However, Beclin1 haploinsufficiency can counteract the effects of cold stress by inhibiting ferroptosis [49]. Clearly, cardiomyocyte ferroptosis is closely related to the occurrence and development of cardiac hypertrophy. In our cultured H9c2 cells, levels of GSH and GPX4 decreased, while levels of MDA, ROS, ferrous ions, and PTGS2 increased in the AngII group. This phenomenon could be reversed by Fer‐1, demonstrating ferroptosis in the cardiac hypertrophy model. Interestingly, we found that TQ alleviated cardiac hypertrophy by inhibiting ferroptosis. Pretreatment of AngII‐induced H9c2 cells with TQ resulted in increased GSH and GPX4 levels, decreased MDA, ROS, and PTGS2 levels, and reduced expressions of cardiac hypertrophy genes ANP, BNP. We show for the first time that TQ can attenuate AngII‐induced H9c2 cell hypertrophy by inhibiting ferroptosis.

Peroxisome proliferator‐activated receptor γ (PPAR‐γ) is a nuclear receptor that plays a crucial role in regulating multiple biological processes such as adipocyte differentiation, insulin sensitivity, energy balance, and inflammatory response [50]. Several studies have shown that PPAR activation has a protective effect on cardiomyocytes. Yamamoto et al. [51] found that PPAR‐γ is present in cardiomyocytes and inhibits cardiomyocyte hypertrophy by regulating the NF‐κB pathway. Piperine can attenuate myocardial hypertrophy and fibrosis by activating the PPAR‐γ/AKT pathway, which is reversed by GW9662 (a specific inhibitor of PPAR‐γ) [52]. In the present study, we found that PPAR‐γ was downregulated in the cardiac hypertrophy model, and TQ pretreatment alleviated cardiac hypertrophy by upregulating PPAR‐γ expression and inhibiting ferroptosis, while GW9662 pretreatment reversed these effects.

Cardiomyocyte apoptosis is closely related to the progression of cardiac hypertrophy. Persistent cardiac hypertrophy can also exacerbate cardiomyocyte apoptosis, leading to cardiac dysfunction and even heart failure [53]. The Bcl‐2 family is a group of proteins closely related to the regulation of apoptosis, with Bcl‐2 representing the anti‐apoptotic protein subfamily and Bax representing the pro‐apoptotic protein subfamily. Bax and Bcl‐2 interact to regulate apoptosis [54]. In this study, pretreatment with TQ significantly upregulated the expression of Bcl‐2 and downregulated the expression of Bax. However, pretreatment with GW9662 significantly reversed the protective effects of TQ. In conclusion, TQ could protect against cardiac hypertrophy by inhibiting apoptosis through upregulating PPAR‐γ.

An increasing number of studies have demonstrated that the PI3K/AKT signalling pathway plays a crucial role in mitigating cardiac hypertrophy [55, 56]. Elevated phosphorylation levels of PI3K and AKT were indicative of the activation of the PI3K/AKT signalling pathway. In the current study, we observed that pretreatment with TQ upregulated the expression levels of phosphorylated PI3K (p‐PI3K) and phosphorylated AKT (p‐AKT). To delve deeper into the mechanism underlying TQ's protective effect, we employed Western blot analysis to assess the phosphorylation status of PI3K and AKT. Our findings revealed that the upregulation of p‐PI3K and p‐AKT induced by TQ pretreatment could be reversed by GW9662 (Figure 8). These results imply that the PPAR‐γ/PI3K/AKT pathway is implicated in the protective role of TQ against cardiac hypertrophy.

In this study, only the PPAR‐γ inhibitor GW9662 was used to investigate the molecular mechanism of TQ's protective effect in vitro and in vivo. For the reliability of the experimental results, it is necessary to use a gene knockout rat model for in vivo verification or PPAR‐γ knockdown cells for in vitro verification in future studies.

**FIGURE 9:**
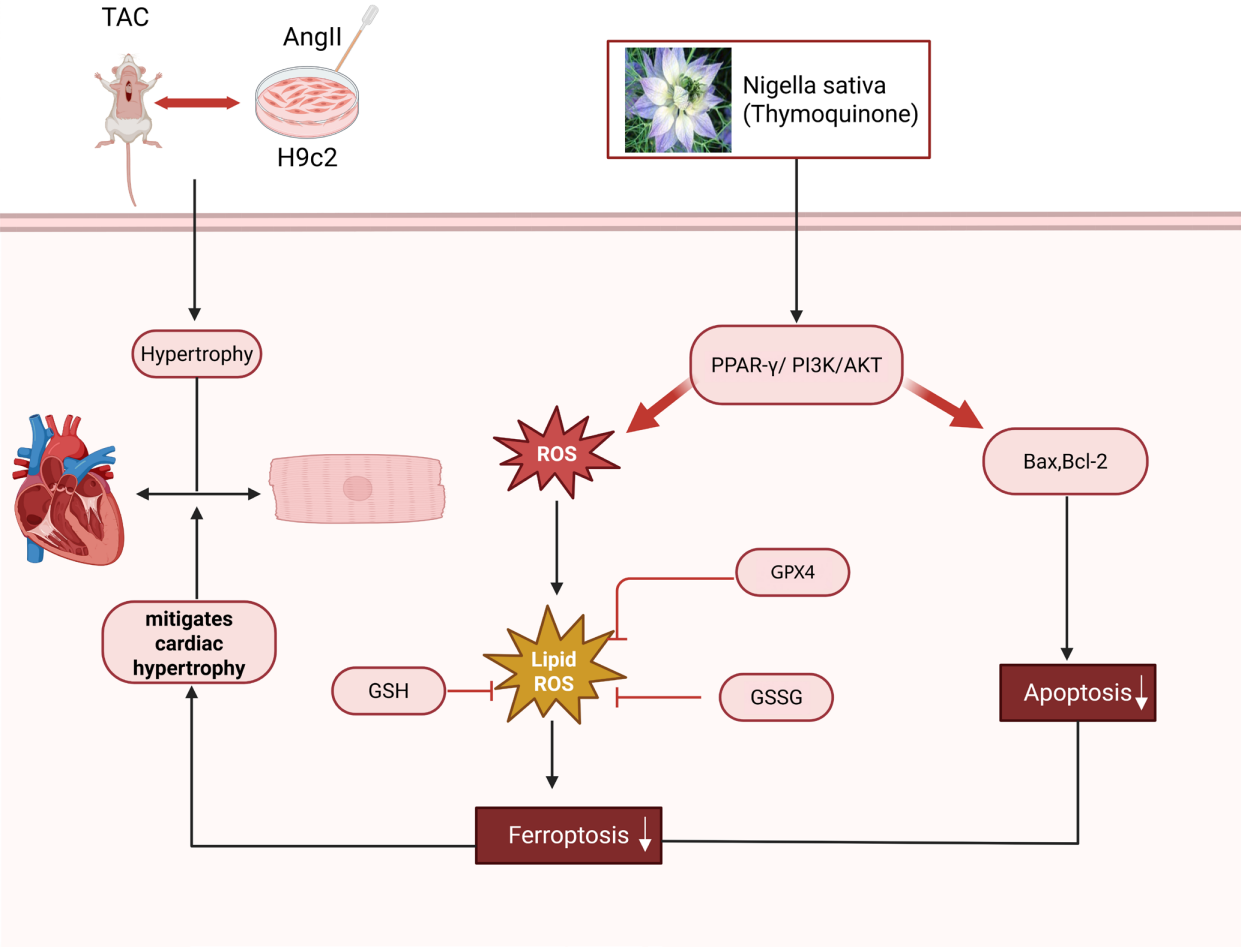
Unravelling the Potential Mechanisms of Thymoquinone in Cardiac Hypertrophy. TQ protects against cardiac hypertrophy via the PPAR‐γ/PI3K/Akt pathway.

In conclusion, the current study has demonstrated that TQ inhibits fibrosis, ferroptosis, and apoptosis through the PPAR‐γ/PI3K/AKT signalling pathway, thereby exerting a protective effect against pressure overload‐induced cardiac hypertrophy (Figure 9). These findings offer valuable insights into the cardioprotective mechanisms of TQ and its potential therapeutic application in the treatment of cardiac hypertrophy.

## Author Contributions

**Rong‐bin Qiu:** conceptualization (equal), writing – original draft (equal). **Zi‐ming Wu:** formal analysis (equal). **Zhi‐qiang Xu:** conceptualization (equal), formal analysis (equal). **Li‐juan Hu:** writing – review and editing (equal). **Shi‐tao Zhao:** data curation (equal). **Rui‐yuan Zeng:** software (equal). **Zhi‐cong Qiu:** conceptualization (equal), software (equal). **Lian‐fen Zhou:** software (equal), visualization (equal). **Song‐qing Lai:** supervision (equal). **Wen‐jun Wang:** project administration (equal). **Li Wan:** funding acquisition (equal).

## Ethics Statement

Animal experiments followed the guidelines of the National Institutes of Health and were authorized by the Animal Experimentation Ethics Committee of the First Affiliated Hospital of Nanchang University (Nanchang, China).

## Consent

The authors have nothing to report.

## Conflicts of Interest

The authors declare no conflicts of interest.

## Data Availability

The original data supporting the conclusions of this paper will be available upon request to any qualified researcher.
